# Efficacy and safety of massage in the treatment of poststroke depression: A systematic review and meta-analysis of protocols

**DOI:** 10.1097/MD.0000000000023908

**Published:** 2021-01-29

**Authors:** Yu Shen, Qiurui Nie, Yajing Zhang, Lijun Xu

**Affiliations:** a Department of Neurology, The Second Affiliated Hospital of Nanchang University; b Jiangxi Provincial People's Hospital, Jiangxi; c College of Acupuncture-Moxibustion and Tuina, Jiangxi University of Traditional Chinese Medicine, Nanchang, China.

**Keywords:** massage, poststroke depression (PSD), protocol, systematic review

## Abstract

**Background:**

Poststroke depression (PSD) is a severe problem; it will significantly increase the mortality of patients after stroke, and affect the quality of life of patients after discharge. For stroke patients without noticeable adverse reactions, massage can effectively improve the patient's mood, thereby treating poststroke depression. But so far, there is still no systematic research to provide reliable evidence that massage can effectively treat poststroke depression. Therefore, the purpose of this study is to comprehensively summarize and evaluate the effectiveness and safety of massage therapy for poststroke depression.

**Methods:**

We conduct a detailed search regardless of publication grade and language status. The search databases include the Web of Science, the Cochrane Library search, EMBASE, PubMed, CNKI, Chinese biomedical literature database, Chongqing VIP, and Wanfang. All randomized controlled trials and cohort studies on massage therapy for poststroke depression are published, as of November 15, 2020. The team consists of 2 experienced researchers who will select the retrieved documents and extract data. Later they used RevMan V.5.3 software for data analysis and data synthesis.

**Results:**

The effectiveness and safety of massage therapy intended for poststroke depression will be subject to a systematic evaluation under this program.

**Conclusion:**

It will be substantiated in this review whether massage therapy is a reliable intervention for poststroke depression by examining the evidence collected.

**INPLASY registration number:**

INPLASY2020110085.

## 1 Introduction

Stroke is a tissue damage caused by a sudden shortage of blood supply to the brain. The cause is ischemia or bleeding. And almost 85% of strokes are ischemic, and 12% are hemorrhagic.^[1]^ In addition to damage to related brain functional areas, stroke can also cause corresponding mental illnesses, including depression, anxiety, cognitive impairment, psychosis, etc.^[2]^ Poststroke depression was discovered by psychiatrists in 1920.^[3]^ In a highly reliable longitudinal study, it was found that the existence of PSD is related to the ability of daily living, social function, cognitive function, and after discharge of stroke patients. Subsequent reissues are obviously related.^[4]^ As a secondary depression, poststroke depression has a high incidence, fatality rate and disability rate. Some studies have found that the incidence of PSD in Portugal is 44.6%, and 4% in the acute phase of stroke patients in the UK The incidence of PSD after 1 month is 28% to 56%.^[5–6]^ Zhang et al^[7]^ studied 91 stroke patients and found that the incidence of PSD at 2 weeks after stroke was 27.47%, of which 88% were mildly depressed, and 12% were moderately depressed. It can be seen that the incidence of poststroke depression is exceptionally high, which should arouse our attention.

At present, the treatment method for poststroke depression is mainly drug therapy. The first randomized, double-blind treatment experiment on PSD was conducted in 1986. Compared with patients receiving a placebo treatment, nortriptyline has a significant effect, which is manifested as a significant reduction in the scores of the depression rating scale.^[8]^ Another double-blind controlled study in 1994 showed that citalopram is having a significant effect.^[9]^ A meta-analysis involving 52 reviews^[10]^ showed that SSRI drugs could significantly reduce the disability rate and neurological deficits of patients when treating depression after stroke and improving the mood of patients. It can be seen that drug treatment has an important position. But we should also know the side effects and risks of drug treatment and economical consumption. Studies have shown that the use of SSRI drugs increases the risk of bleeding complications and makes the elderly more likely to fall.^[11]^ There are also epidemiological studies that show that SSRI is also indispensable to patients’ subsequent strokes.^[12–13]^ Therefore, there are still a large number of studies exploring the treatment of poststroke depression with SSRI, including the types of drugs, dosages, start and end times of use, and so on. So, there are still many unsolvable problems in the drug treatment of poststroke depression.

In summary, we should find a safer, cheaper, and effective treatment intervention for patients with poststroke depression. According to research, massage can effectively improve mood, create a feeling of pleasure, and reduce the occurrence of major adverse events in stroke patients.^[14–17]^ Massage is widely used as a method, as a complementary and alternative therapy for the treatment of many diseases.^[15]^

First of all, massage helps promote blood circulation and strengthen immunity, so as to achieve the purpose of preventing and curing diseases. Secondly, existing studies have shown that the comfort generated by local massage can be transmitted to the central nervous system after being felt by the surrounding receptors, so that our brain can produce a feeling of relaxation, tranquility and calm, and let the whole body be in a relaxed state.^[16–19]^ Finally, massage therapy is easy to operate, and operators with basic knowledge can perform massage therapy in a few months. And there are no apparent adverse reactions, and the price is low. So far, many studies have shown that massage can effectively relieve symptoms related to depression.^[20–21]^

As far as we know, there is no systematic review on whether massage therapy for poststroke depression is safe and effective. Therefore, this program aims to evaluate the efficacy of poststroke massage for depression comprehensively.

## 2 Methods

### 2.1 Study registration

The registration number is INPLASY2020110085. We will implement this Agreement in strict accordance with the preferred reporting items in the guidelines for Systematic Review and Meta-Analysis (PRISMA-P) statements.^[22]^

### 2.2 Inclusion criteria for study

#### 2.2.1 Type of studies

All RCTs and cohort studies of massage therapy for poststroke depression will be included, regardless of language or publication status. However, animal tests, case reports, empirical reports, and reviews will not be included.

#### 2.2.2 Types of participants

It will include all patients with poststroke depression regardless of stroke type, race, sex, education level or age.

#### 2.2.3 Types of interventions

##### 2.2.3.1 Experimental interventions

The experimental group only received massage, massage, pressure, foot reflex treatment. There is no limit to the frequency, intensity, duration, location, or type of treatment.

##### 2.2.3.2 Control interventions

The control group could receive any therapy other than massage, including drugs, acupuncture, psychotherapy, placebo, etc.

#### 2.2.4 Types of outcome measures

##### 2.2.4.1 Primary outcomes

- Self-rating Anxiety Scale (SAS),
- Self-rating Depression Scale (SDS),
- Hamilton Depression Rating Scale (HAM-D).

##### 2.2.4.2 Secondary outcomes

Adverse events (including patient withdrawal or death due to irresistible factors)

### 2.3 Search methods

#### 2.3.1 The primary source of data

We will retrieve all RCTs and cohort studies of massage therapy for poststroke depression from the following database, including the Web of Science, the Cochrane Library search, EMBASE, PubMed, CNKI, Chinese biomedical literature database, Chongqing VIP, and Wanfang, by November 15, 2020. Table 1 describes in detail the retrieval strategies adopted by PubMed.

**Table 1:** Search strategy for PubMed database.

Number	Search items
#1	randomized controlled trial [pt]
#2	controlled clinical trial [pt]
#3	randomized [tiab]
#4	clinical trials as topic [mesh: noexp]
#5	randomly [tiab]
#6	trial [ti]
#7	OR/#1–#6
#8	animals [mh] NOT humans [mh]
#9	#7 NOT #8
#10	Stroke[Mesh]
#11	Cerebral Stroke[All Fields)
#12	Apoplexy[All Fields)
#13	Cerebrovascular Apoplexy[All Fields)
#14	Cerebrovascular Accident[All Fields)
#15	OR/#10–#14
#16	Depression[Mesh]
#17	Depressions[All Fields)
#18	Depressive Symptom[All Fields)
#19	Emotional Depression[All Fields)
#20	OR/#16–#19
#21	Massage[Mesh]
#22	Massage Therapy[All Fields)
#23	Zone Therapy[All Fields)
#24	Acupressure[All Fields)
#25	Manipulate[All Fields)
#26	Tuina[All Fields)
#27	Anmo[All Fields)
#28	OR/#21–#27
#29	#9 AND #15 AND #20AND #28

#### 2.3.2 Search of other resources

We will retrieve some uncompleted or unpublished trial data from the China Clinicaltrials registry and clinicaltrials.gov.

### 2.4 Data collection and analysis

#### 2.4.1 Literature selection

**Figure 1:**
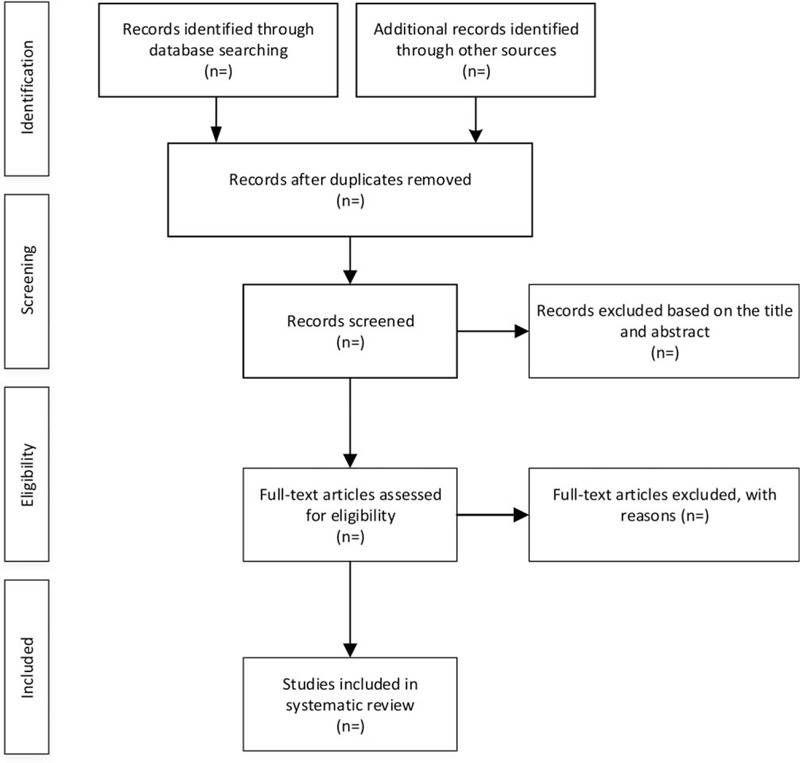
Flow diagram of study selection process.

First, all the literature will be imported into the EndNote X9 software, and all duplicate literature will be deleted. Secondly, the title and abstract will be rigorously reviewed by researchers YS and YZ to remove irrelevant literature. Third, they should read the full text and decide whether to include the literature. Finally, 2 researchers (YS and YZ) will cross-check. If there is any disagreement, the third researcher (LX) will participate in the discussion and solve it. Figure 1 shows a flow chart of literature screening.

#### 2.4.2 Data extraction and management

The 2 authors will extract the following data: journal, author information, title, publication time, participant characteristics, sample size, interventions, study methods, primary and secondary outcome measures, and any adverse events, respectively. A third author will step in to address any potential differences.

### 2.5 Risk of bias assessment

The quality of the trials will be examined using the Cochrane Bias risk Assessment tool, respectively, by YS and YZ. The extracted details include the random sequence generation, the blindness of result evaluation, the blindness of participants and personnel, the concealment of allocation, the reporting of selective results, and the incomplete result data, etc., which are divided into 3 levels: fuzzy, low, and high. If the relevant research is ambiguous, we will contact the author to get the information we want. If there is any dispute, an informed decision will be made with the assistance of the third investigator (LX).

### 2.6 Data synthesis

The RevMan 5.3 software will be used for data analysis. When the measured results were dichotomous, a 95% confidence interval risk ratio (RR) was used. With continuous variables as the measurement results, the weighted average difference (WMD) with CI of 95% was selected when the measurement tools were the same. Otherwise, a 95% CI standard mean difference (SMD) is used.

### 2.7 Heterogeneity assessment

I^2^ was calculated to evaluate heterogeneity across studies. *I*^2^ < 25% considered homogeneity; 25% ≤ *I*^2^ < 50% considered low heterogeneity; 50% ≤ *I*^2^ < 75% considered moderate heterogeneity; and *I*^2^ ≥ 75% considered substantial heterogeneity.^[23]^ If the data is homogeneous or low heterogeneity, use the fixed effects model for analysis; if the data has moderate or significant heterogeneity, use the random effects model.^[24]^ All analyses were conducted using RevMan 5.3 software (Copenhagen: The Nordic Cochrane Centre, The Cochrane Collaboration, 2014).

### 2.8 Subgroup analysis

When significant heterogeneity exists among the tests involved, we will take into account factors such as massage mode, poststroke depression severity, course of the disease, stroke type, sample size, and so on for subgroup analysis.

### 2.9 Sensitivity analysis

Sensitivity analysis will be used to test the quality of the literature to exclude low-quality literature and to ensure the stability and reliability of the conclusions drawn from the meta-analysis.

### 2.10 Assessment of reporting biases

When the number of articles exceeded 10, the funnel plot was used to analyze whether there was publication bias. If there is an asymmetric funnel plot, the Egger check will be used to study the causes of publication bias.

### 2.11 Quality of evidence

The Grade Profiler 3.6 software will be used by the 2 researchers to measure the quality of evidence for the outcome measures, including high evidence, intermediate evidence, low evidence, and very low evidence.

### 2.12 Ethics and dissemination

The study is not related to the patient's personal information and therefore does not require ethical approval. The results are expected to be published in peer-reviewed journals.

## 3 Discussion

PSD often manifests as anxiety, depression, and related psychiatric symptoms after stroke, which will not only affect the later recovery of stroke patients, but also increase the risk of stroke recurrence.^[12–13]^ At present, drug therapy is considered to be the main effective method to treat PSD. However, drug treatment may also produce various side effects such as bleeding, and the drugs are expensive. As a common auxiliary therapy, massage can effectively relieve symptoms related to depression, thereby improving the quality of life of stroke patients.^[18–21]^ In addition, it also has the advantages of low price, high safety, easy operation, and minimal side effects, which makes massage therapy an ideal choice for patients with insomnia after stroke. So far, the efficacy of massage therapy in treating depression after stroke remains to be confirmed.

Therefore, it is hoped that this study can provide some reliable evidence and valuable reference for the treatment of poststroke depression through massage therapy. Finally, a good treatment plan was found for poststroke depression.

However, this study may have some limitations: First, due to different massage methods, including different techniques and parts, there may be some heterogeneity. Secondly, this study does not restrict language, which may lead to language bias.

## Author contributions

**Conceptualization:** Yu Shen, Qiurui Nie, Yajing Zhang, Xu Lijun.

**Data curation:** Yu Shen, Yajing Zhang.

**Formal analysis:** Yu Shen, Yajing Zhang.

**Funding acquisition:** Yu Shen, Yajing Zhang.

**Methodology:** Qiurui Nie, Yajing Zhang.

**Software:** Qiurui Nie, Yajing Zhang.

**Writing – original draft:** Yu Shen.

**Writing – review & editing:** Yu Shen, Xu Lijun.

## Abbreviations

Abbreviations: CI = confidence interval, HAM-D = Hamilton Depression Rating Scale, PSD = poststroke depression, RCTs = randomized controlled trials, SAS = Self-rating Anxiety Scale, SDS = Self-rating Depression Scale.
